# Do assets explain the relation between race/ethnicity and probable depression in U.S. adults?

**DOI:** 10.1371/journal.pone.0239618

**Published:** 2020-10-02

**Authors:** Catherine K. Ettman, Gregory H. Cohen, Salma M. Abdalla, Sandro Galea

**Affiliations:** 1 Office of the Dean, Boston University School of Public Health, Boston, Massachusetts, United States of America; 2 Department of Health Services, Policy, and Practice, Brown University School of Public Health, Providence, Rhode Island, United States of America; 3 Department of Epidemiology, Boston University School of Public Health, Boston, Massachusetts, United States of America; 4 Department of Epidemiology, Columbia Mailman School of Public Health, NYC, New York, United States of America; University of Arkansas for Medical Sciences, UNITED STATES

## Abstract

Depression is a leading cause of disability in the U.S. across all race/ethnicity groups. While non-Hispanic Black and Hispanic persons have worse physical health on most indicators than non-Hispanic White persons, the literature on the association between race/ethnicity and rates of depression is mixed. Given unequal distribution of assets across racial/ethnic groups, it is possible that social and economic differences may explain differential rates of depression across race/ethnicity groups. Using National Health and Nutrition Examination Survey (NHANES) data from 2007–2016, we constructed a nationally representative sample of 26,382 adults over 18 years old (11,072 non-Hispanic White, 5,610 non-Hispanic Black, 6,981 Hispanic, and 2,719 Other race). We measured symptoms of depression using the Patient Health Questionnaire-9 (PHQ-9), with a score of 10 or more indicating probable depression. We identified three kinds of assets: financial assets (income), physical assets (home ownership), and social assets (marital status and education). We estimated the weighted prevalence of probable depression across race/ethnicity groups, odds ratios controlling for assets, and predicted probabilities of probable depression across race/ethnicity and asset groups. Three results contribute to our understanding of the differences in probable depression rates between race/ethnicity groups: 1) Non-Hispanic Black and Hispanic persons had a higher weighted prevalence of probable depression in the U.S. than non-Hispanic White persons. In models unadjusted for assets, non-Hispanic Black and Hispanic persons had 1.3 greater odds of probable depression than non-Hispanic White persons (p<0.01). 2) We found an inverse relation between assets and probable depression across all race-ethnicity groups. Also, non-Hispanic Black and Hispanic persons had fewer assets than non-Hispanic Whites. 3) When we controlled for assets, non-Hispanic Black and Hispanic persons had 0.8 times lower odds of probable depression than non-Hispanic White persons (p<0.05). Thus, when holding assets constant, minorities had better mental health than non-Hispanic White persons in the U.S. These three findings help to reconcile findings in the literature on race/ethnicity and depression. Given vastly unequal distribution of wealth in the U.S., it is not surprising that racial minorities, who hold fewer assets, would have an overall larger prevalence of mental illness, as seen in unadjusted estimates. Once assets are taken into account, Black and Hispanic persons appear to have better mental health than non-Hispanic White persons. Assets may explain much of the relation between race/ethnicity group and depression in the U.S. Future research should consider the role of assets in protecting against mental illness.

## Introduction

Depression is a common mood disorder in the U.S. affecting all race/ethnicity groups. During any given 2-week period, 8.1% of American adults report symptoms consistent with depression [1]. Depression is costly and is associated with reduced physical health across multiple dimensions. For example, persons with depression have increased levels of obesity, coronary heart disease, and lower life expectancy [2–4].

While racial minorities have demonstrably worse health indicators across a range of physical health indicators, the association between race/ethnicity and depression is more complicated [5–8]. The literature on depression and race/ethnicity is divided. For example, some studies suggest that Black persons have a higher prevalence of depression [1, 9–11] while others have shown that Black persons have a lower prevalence of depression than their White counterparts [12–17], resulting in what has been called the Black-White depression paradox [18]. In part, the difference in prevalence of depression outcomes appears to depend on whether studies adjusted for other factors or not; for example, in a study of adults ages 54 to 65 years old, Dunlop et al. found a higher prevalence of 12-month major depression among African American persons than White persons [11]. Once they adjusted for sociodemographic, health, and economic profiles, African American persons exhibited a lower adjusted prevalence of 12-month major depression than their White counterparts. Definitions, and types, of depression also matter, with studies showing different relations depending on the chronicity and severity of depression [19]. For example, Breslau et al. found that Non-Hispanic Black persons had significantly lower prevalence of major depression but no significant difference in the prevalence of dysthymic disorder (a lower severity measure of depression) than non-Hispanic White persons [13]. Williams et al. found that both African American Black persons and Caribbean Black persons had a lower lifetime prevalence of major depressive disorder (MDD) than non-Hispanic White persons, but a higher risk of the persistence of MDD than non-Hispanic White persons [16].

The literature on depression among other racial minorities is limited. Depression has been documented to be both higher and lower in Hispanic persons than non-Hispanic White persons [11–13] and lower in non-Hispanic Asian persons than non-Hispanic White persons [1, 12]. According to the National Center for Health Statistics, during 2013–2016, non-Hispanic Asian adults experienced the lowest prevalence of depression (3.1%) relative to Hispanic adults (8.2%), non-Hispanic White adults (7.9%), and non-Hispanic Black adults (9.2%) [1]. However, other studies have found different relations among the race/ethnicity groups. Gavin et al. found that Black, Latino, and Asian persons had lower 12-month MDD than White persons [12]. Indigenous Americans report higher rates of depressive disorders than White persons [20] though they are frequently under-studied. Last, there is heterogeneity within racial/ethnic groups that are often grouped together in analyses (for example, differences between Caribbean and American-born Black persons, between Asian-subgroups, and between Hispanic-subgroups, among others).

Thus, the evidence comparing depression prevalence among racial and ethnic minorities and non-Hispanic White persons in the United States appears to be mixed [11, 13, 16, 19]. What may explain these different findings?

It is plausible that assets—or lack thereof—may contribute to racial and ethnic differences in depression rates. Having assets improves mental health [21–23], and higher income is associated with lower prevalence of depression [1, 24]. Having higher income provides access to material resources, mitigates the effects of financial and other stressors, and serves as a proxy for greater social status, power, and authority in the workplace, each of which is associated with better mental health [25, 26]. Having more education, being married, and owning a home are all associated with lower prevalence of depression [27, 28].

Racial and ethnic minorities in the United States have long had less access to assets primarily due to biased laws, policies, and systems that made it challenging for non-White persons to buy homes, attend institutions of higher learning, or acquire assets [29]. Non-Hispanic Black persons, for example, have lower assets and access to fewer resources than non-Hispanic White persons in the U.S. [29, 30]. Therefore, access to assets may be a large part of what drives the White-Black differences in depression prevalence in the United States. The most direct evidence for this line of thinking comes from Peplinski et al. who studied depressive symptoms and race, controlling for education, income, and marital status in adults ages 45–84 using the Multi-Ethnic Study of Atherosclerosis. They found that Black and Hispanic persons had a higher prevalence of depressive symptoms than their White counterparts, and posited that significant disparities in socioeconomic status (SES) likely contributed to higher rates of depression for those groups [10].

We are not aware of existing analyses that have explored whether assets explain the prevalence and odds of current probable depression across different racial/ethnic groups in U.S. adults ages 18 and older. Therefore, aiming to understand the role of assets in shaping racial/ethnic differences in depression prevalence, we explored the relation between depression, race/ethnicity, and assets in a nationally representative sample of U.S. adults ages 18 and older. We aimed 1) to estimate the prevalence of probable depression in a nationally representative sample, stratified by self-identified race/ethnicity group and 2) to understand how assets might influence the prevalence of probable depression across race/ethnicity groups. We considered three types of assets: financial assets (household income), physical assets (home ownership), and social assets (marital status and education).

## Materials and methods

The National Health and Nutrition Examination Survey (NHANES) is an annual survey conducted by the U.S. Government that collects health and household information about a representative sample of the U.S. population. This study used data from the following waves of NHANES: 2007–2008, 2009–2010, 2011–2012, 2013–2014, and 2015–2016. Together, 50,588 participants were interviewed from 2007–2016. The study sample excluded persons below the age of 18 (n = 19,864) and persons missing depression data (n = 4,342). The final sample included 26,382 adults above age 18 (11,072 non-Hispanic White, 5,610 non-Hispanic Black, 6,981 Hispanic, and 2,719 Other race).

### Variables

Probable depression was measured using the Patient Health Questionnaire (PHQ-9) with a cut-off score of 10. The PHQ-9 is a clinically validated instrument for the assessment of Diagnostic and Statistical Manual of Mental Disorders (DSM)-IV major depression, performing with a sensitivity and specificity of 88% for clinical diagnosis when using the PHQ-9 cutoff score of 10 [31, 32]. Participants were guided through a computer assisted personal interview that asked about behaviors, thoughts, and feelings over the last two weeks. Participants were scored based on their responses, with scores ranging from 0 to 27. Scores of 10 or greater were defined as having past month probable depression and scores below 10 were defined as not having probable depression.

Race/ethnicity was defined as a self-reported, mutually exclusive categorical variable: “Non-Hispanic White,” “Non-Hispanic Black,” “Hispanic,” or “Other race.” “Other race” included multiracial and non-Hispanic Asian. Any person reporting more than one race was included in the “Other race” category. Household income was divided into four categories, roughly following the interquartile range for the categorical variables of income: $0-$19,999; $20,000-$44,999; $45,000-$74,999; ≥$75,000. Home ownership was defined as a binary variable. Participants were asked “Is this {mobile home/house/apartment} owned, being bought, rented, or occupied by some other arrangement by {you/you or someone else in your family}?” Participants who answered “owned or being bought” were defined as “home owners” and “rented or occupied by some other arrangement” were defined as “home renters.” Marital status was defined as a categorical variable: “Married,” “Widowed, divorced, or separated,” “Never married,” or “Living with partner.” Educational attainment was defined as a categorical variable with four levels: “Less than high school graduate,” “High school graduate or GED,” “Some college,” and “College graduate or more.” Gender was a binary variable defined as “male” or “female.” Age was defined as a categorical variable by the following three groups: 18–39, 40–59, ≥60. These groupings were consistent with age categories recommended by the NHANES analytic guidelines [33]. Household size was defined a numerical variable from 1 to 7. Households with more than 7 persons were categorized as 7 to maintain anonymity. NHANES cohort wave was defined as a categorical variable with the 2-year cycle in which the person was sampled.

Survey weights were used to calculate effect estimates that are representative of the U.S. population. To obtain stable prevalence measures for racial minorities in the data collection process, NHANES oversampled Hispanic persons, non-Hispanic Black persons, and non-Hispanic non-black Asian persons, among others. To account for oversampling, survey non-response, and post-stratification, NHANES included survey weights assigned to each participant. We used the MEC survey weights to produce results that are representative of the U.S. non-institutionalized civilian population.

### Analysis

First, we summarized frequencies and weighted percentages of the demographic characteristics of the total study sample. Second, we calculated the frequency and estimated the weighted prevalence of persons with probable depression across demographic and asset groups. We conducted two-tailed chi-square analysis to determine if there was a significant difference in the prevalence of probable depression within groups at the 5% significance level. Third, we summarized frequencies and weighted percentages of assets across race/ethnicity groups. Fourth, we estimated unadjusted and adjusted odds ratios of probable depression, adding in demographic and asset variables in a forward stepwise fashion. Model 1 included the relation between probable depression and race/ethnicity only. Models 2 through 8 included the following variables, in the following order: race/ethnicity, gender, age, educational attainment, marital status, home ownership, and household income. The final model, Model 8, also adjusted for household size. All models adjusted for NHANES cohort wave. Fifth, we estimated and graphed the weighted prevalence of probable depression by race/ethnicity and asset categories: financial assets (household income), social assets (educational attainment and marital status), and physical assets (home ownership). We graphed them across non-Hispanic White, non-Hispanic Black, and Hispanic groups; we did not include the Other race category because of heterogeneity of persons and outcomes within this group. Sixth, we estimated the predicted probabilities of depression across race/ethnicity using unadjusted (Model 1) and fully adjusted (Model 8) models. STATA software version 15.1 was used for all analyses (College Station, TX: StataCorp LP).

## Results

Table 1 shows that 7.9% of the U.S. population had probable depression. Twice as many women as men had probable depression (10.1% v. 5.7%). Persons ages 40–59 years had the highest prevalence of probable depression (9.2%) relative to persons ages 18–39 (7.6%) and ages ≥60 (6.7%). Non-Hispanic Black (9.8%) and Hispanic (9.2%) persons had higher prevalence of probable depression than non-Hispanic White persons (7.5%) or persons of Other race (7.2%). Persons with less than a high school degree had the highest prevalence of probable depression (13.1%) relative to persons with high school degrees or GEDs (9.0%), some college (8.3%), or college degrees or more (3.6%). Persons who were married had the lowest prevalence of probable depression (5.4%) relative to persons who were widowed, divorced, or separated (13.5%), never married (9.4%) or living with partners (10.0%). Lower income was associated with higher prevalence of probable depression. Persons with a household income of $0-$19,999 had the highest prevalence of probable depression (15.8%) relative to persons with $20,000-$44,999 (9.6%), $45,000-$74,999 (6.2%), or ≥$75,000 (3.5%) in household income. Home owners had lower weighted prevalence of probable depression (6.4%) than home renters (11.1%). All asset and demographic groups had between-group differences in probable depression that were statistically significant at p<0.001.

**Table 1:** Characteristics in study sample (N = 26,382).

	Total		Probable depression		
Characteristic	N	%	N	%	p-value
**Total**	26,382		2,408	7.9	
**Gender**					
Male	13,018	48.9	861	5.7	<0.001
Female	13,364	51.1	1,547	10.1	
**Age (y)**					
18–39	9,715	38.2	814	7.6	
40–59	8,232	36.3	916	9.2	<0.001
≥60	8,435	25.5	678	6.7	
**Race**					
Non-Hispanic White	11,072	67.5	1,004	7.5	<0.001
Non-Hispanic Black	5,610	11.2	515	9.8	
Hispanic	6,981	14.2	725	9.2	
Other race	2,719	7.1	164	7.2	
**Education**					
Less than high school graduate	6,732	17.0	905	13.1	<0.001
High school graduate or GED	6,542	23.6	602	9.0	
Some college	7,325	30.8	666	8.3	
College graduate or more	5,764	28.7	234	3.6	
**Marital status**					
Married	12,783	55.2	809	5.4	<0.001
Widowed, divorced, or separated	5,591	18.5	792	13.5	
Never married	4,627	18.3	491	9.4	
Living with partner	1,995	8.0	218	10.0	
**Household income**					
$0-$19,999	6,431	18.4	1,019	15.8	<0.001
$20,000-$44,999	7,560	26.6	714	9.6	
$45,000-$74,999	4,588	21.2	292	6.2	
≥$75,000	5,868	33.8	202	3.5	
**Home ownership**					
Rent	10,255	31.3	1,261	11.1	<0.001
Own	15,832	68.8	1,120	6.4	

Note: The National Health and Nutrition Examination Survey (NHANES) 2007–2016. Frequencies (N) are unweighted. Percentages (%) are weighted. Missing values: education (n = 19), marital status (n = 1,386), income (n = 1,935), home ownership (n = 295). Two-tailed chi-square analysis done for significance testing. GED is the general education diploma. Probable depression defined by Patient Health Questionnaire-9 (PHQ-9) cutoff of ≥10.

Table 2 shows the distribution of assets by race/ethnicity. Persons of Other race had the highest level of college graduates (41.1%) followed by non-Hispanic White (32.9%), non-Hispanic Black (17.2%), and Hispanic persons (11.5%). Non-Hispanic White persons were most likely to be married (59.2%), followed by Other race (58.7%), Hispanic (51.2%), and non-Hispanic Black (33.4%) persons. Non-Hispanic White persons were most likely to have a household income of $75,000 or more (39.5%), followed by Other race (35.2%), non-Hispanic Black (19.5%) and Hispanic (16.2%) persons. Non-Hispanic White persons were most likely to own a home (75.2%), followed by Other race (59.5%), Hispanic (50.1%) and non-Hispanic Black (47.3) persons.

**Table 2:** Distribution of assets in study sample of U.S. adults by race/ethnicity (N = 26,382).

	Non-Hispanic White			Non-Hispanic Black				Hispanic				Other race			
Characteristics	N	%		N		%		N		%		N		%	
**Education**															
Less than high school graduate	1,809	11.4		1,356		21.7		3,165		41.3		402		13.5	
High school graduate or GED	2,853	23.7		1,599		27.8		1,583		23.3		507		17.2	
Some college	3,439	32.1		1,750		33.3		1,483		23.9		653		28.2	
College graduate or more	2,967	32.9		902		17.2		741		11.5		1,154		41.1	
**Marital status**																
Married	5,949	59.2		1,867		33.4		3,446		51.2		1,521		58.7	
Widowed, divorced, or separated	2,498	18.7		1,414		23.7		1,342		15.9		337		13.2	
Never married	1,555	15.3		1,535		33.5		990		19.4		547		21.3	
Living with partner	704	6.7		428		9.5		718		13.5		145		6.8	
**Household income**																
$0-$19,999	2,511	14.7		1,597		30.1		1,810		27.4		513		18.1	
$20,000-$44,999	3,080	24.2		1,635		31.4		2,240		36.0		605		24.7	
$45,000-$74,999	1,927	21.7		959		19.0		1,230		20.4		472		21.9	
≥$75,000	3,041	39.5		980		19.5		963		16.2		884		35.2	
**Home ownership**																
Rent	3,141	24.9		2,779		52.7		3,195		49.9		1,140		40.5	
Own	7,867	75.2		2,778		47.3		3,665		50.1		1,522		59.5	

Note: The National Health and Nutrition Examination Survey (NHANES) 2007–2016. Frequencies (N) are unweighted. Percentages (%) are weighted to the U.S. population. Missing values: education (n = 19), marital status (n = 1,386), household income (n = 1,935), home ownership (n = 295). GED is the general education diploma.

Table 3 shows step-wise multivariable logistic regressions with unadjusted and adjusted models. The unadjusted model (Model 1) shows that Non-Hispanic Black and Hispanic persons had 1.3 times the odds of probable depression (p<0.001) relative to non-Hispanic White persons, with no significant difference between depression in non-Hispanic White persons and Other race. The effect estimates for Hispanic and non-Hispanic Black persons remained stable until Model 4 when we added in educational attainment. In Model 4, when we controlled for race, age, gender, and educational attainment, there was no significant difference in odds of probable depression across race/ethnicity. Model 5, which additionally controls for marital status, also showed no significant difference in probable depression between race/ethnicity groups. In Model 6, we added as a control home ownership; we found that non-Hispanic Black persons had 0.89 times lower odds and Hispanic persons had 0.85 times lower odds of having probable depression relative to white persons (p<0.1). In Model 7, we controlled for household income. In our final model, Model 8, we adjusted for race, gender, age, education, marital status, home ownership, household income, and household size. The final model showed that non-Hispanic Black persons had 0.8 times the odds (p<0.05) and Hispanic persons had 0.8 times the odds (p<0.01) of probable depression relative to non-Hispanic White persons, controlling for assets and demographic variables.

**Table 3:** Multivariable logistic regression of probable depression in U.S. adults with variables added in forward stepwise fashion (N = 26,382).

Variables	Model 1	Model 2	Model 3	Model 4	Model 5	Model 6	Model 7	Model 8
Non-Hispanic Black	1.3***	1.3***	1.3***	1.1	1.0	0.9*	0.9**	0.8**
Hispanic	1.3***	1.3***	1.3***	0.9	0.9	0.8*	0.8**	0.8***
Other race	1.0	1.0	1.0	1.0	1.0	1.0	1.0	0.9
Female		1.9***	1.9***	1.9***	1.9***	1.8***	1.8***	1.8***
Age 18–39 years			1.2*	1.3***	1.3***	1.2*	1.4***	1.3**
Age 40–59 years			1.4***	1.6***	1.7***	1.7***	1.9***	1.9***
Less than high school graduate				4.4***	4.0***	3.9***	2.6***	2.5***
High school grad or GED				2.8***	2.7***	2.6***	2.0***	1.9***
Some college				2.4***	2.2***	2.2***	1.8***	1.8***
Widowed, divorced, or separated					2.3***	2.1***	1.7***	1.8***
Never married					1.8***	1.7***	1.4***	1.4***
Living with partner					1.7***	1.6***	1.3***	1.3***
Home renter						1.4***	1.1	1.1
$0-$19,999							3.4***	3.5***
$20,000-$44,999							2.2***	2.2***
$45,000-$74,999							1.5***	1.6***

*** p<0.01

** p<0.05

* p<0.1. Note: NHANES 2007–2016. All models control for NHANES cohort wave and Model 8 controls for household size. Referent categories: non-Hispanic White, male, age ≥60, college degree or more, married, home owner, ≥$75,000. GED is the general education diploma. Probable depression defined by Patient Health Questionnaire-9 (PHQ-9) cutoff of ≥10.

**Fig 1:**
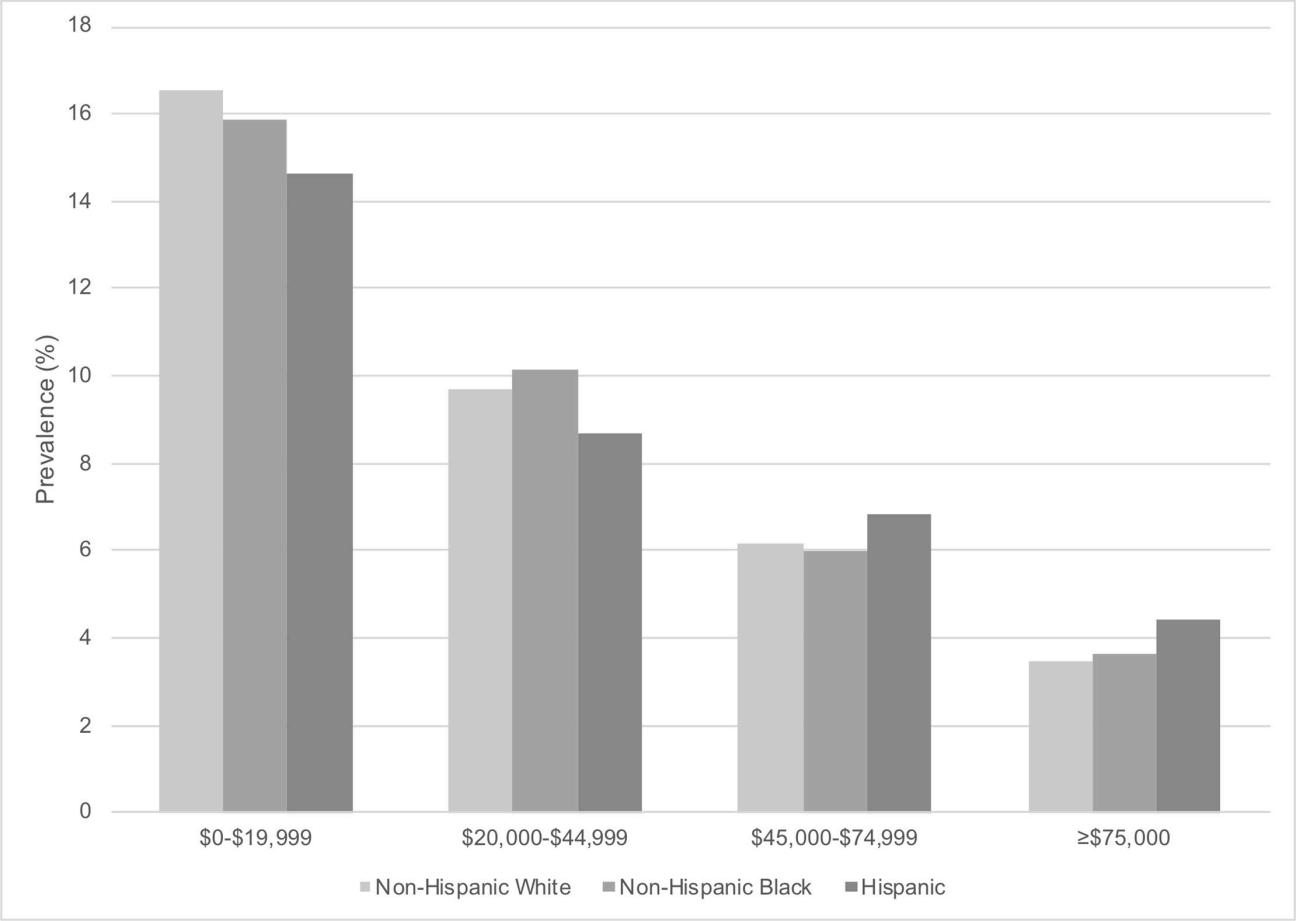
Weighted prevalence of probable depression by race/ethnicity and household income in U.S. adults (N = 26,382). The National Health and Nutrition Examination Survey (NHANES) 2007–2016. Missing values for household income (n = 1,935). Weighted to the U.S. population. Probable depression defined by Patient Health Questionnaire-9 (PHQ-9) cutoff of ≥10.

Fig 1 shows the prevalence of probable depression by race/ethnicity and household income weighted to the U.S. population. Prevalence of probable depression was higher with lower income across all race/ethnicity groups. Among persons in the highest household income category with ≥$75,000, prevalence of probable depression ranged from 3.4% (non-Hispanic White) to 3.7% (non-Hispanic Black) to 4.4% (Hispanic); among persons with the lowest household income category from $0-$19,999, prevalence of probable depression ranged from 14.6% (Hispanic) to 15.9% (non-Hispanic Black) to 16.5% (non-Hispanic White).

**Fig 2:**
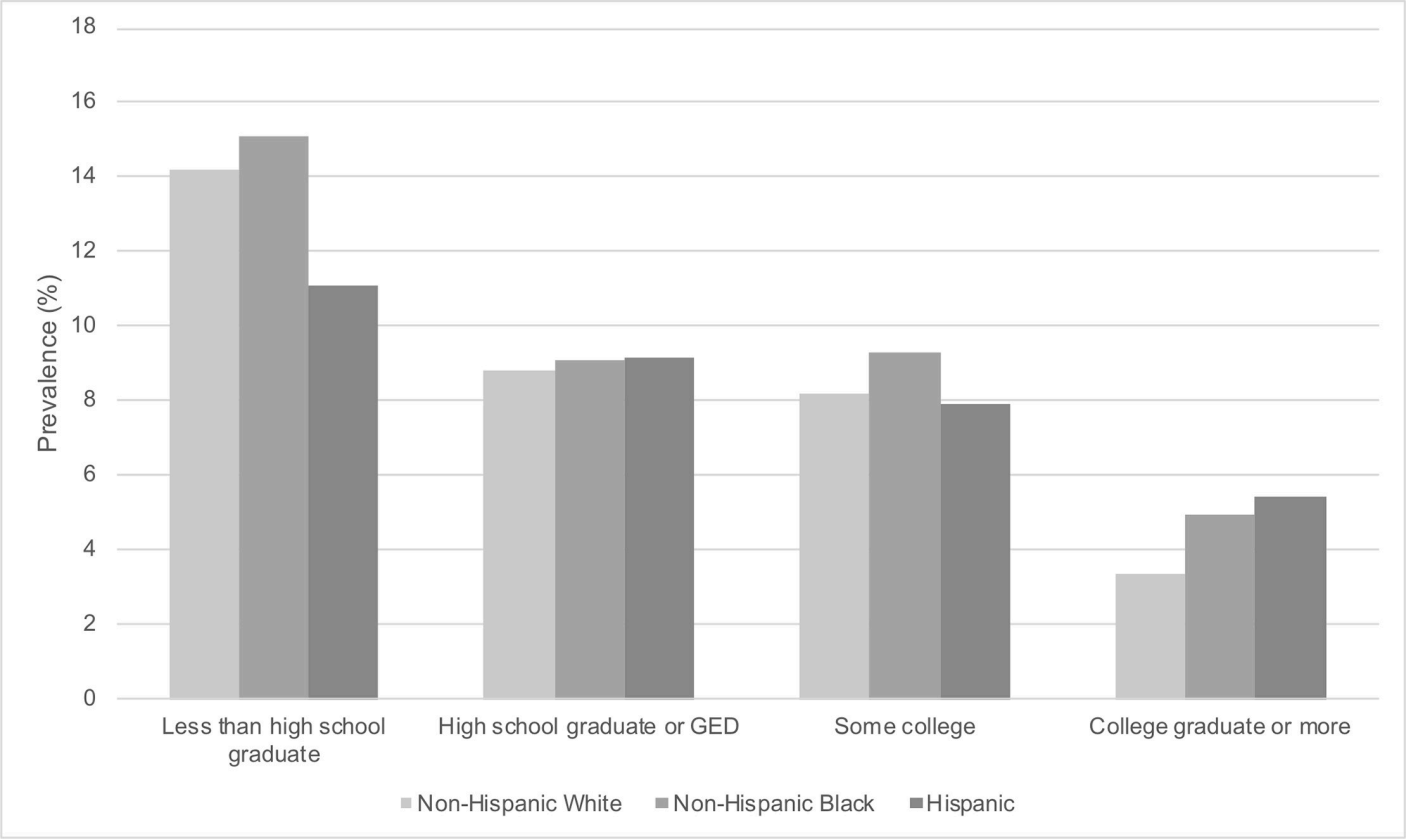
Weighted prevalence of probable depression by race/ethnicity and educational attainment in U.S. adults (N = 26,382). The National Health and Nutrition Examination Survey (NHANES) 2007–2016. Missing values for education (n = 19). Weighted to the U.S. population. GED is the general education diploma. Probable depression defined by Patient Health Questionnaire-9 (PHQ-9) cutoff of ≥10.

Fig 2 shows the weighted prevalence of probable depression by race/ethnicity and educational attainment. Prevalence of probable depression was highest among persons with less than a high school degree across all race/ethnicity groups; prevalence of depression was lowest among persons with a college degree or more across all race/ethnicity groups. Among persons with less than a high school degree, prevalence of probable depression ranged from 11.1% (Hispanic) to 15.07% (non-Hispanic Black) to 14.18% (non-Hispanic White). Among persons with a college degree or more, prevalence of probable depression ranged from 5.42% (Hispanic) to 4.94% (non-Hispanic Black) to 3.38% (non-Hispanic White).

**Fig 3:**
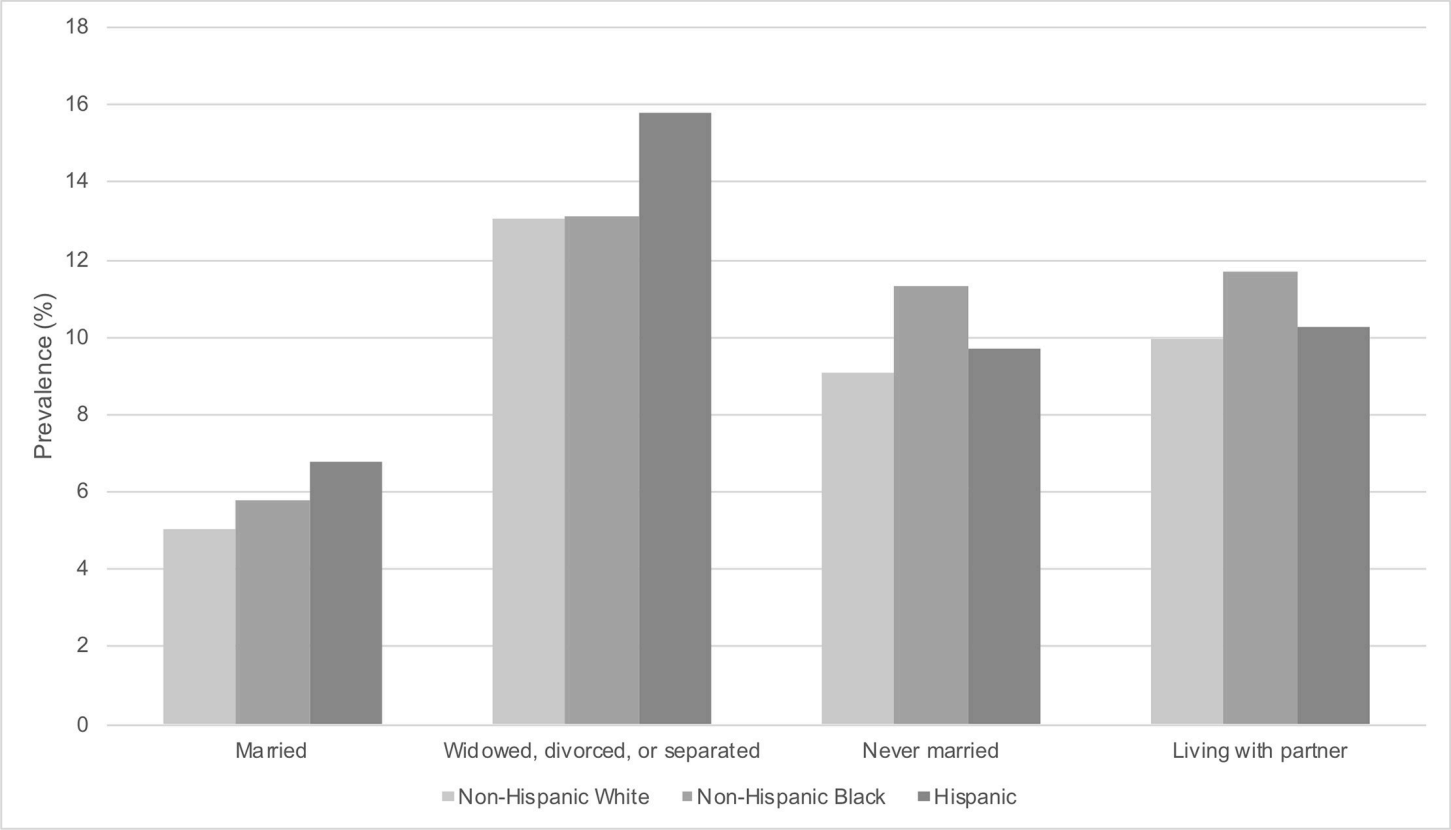
Weighted prevalence of probable depression by race/ethnicity and marital status in U.S. adults (N = 26,382). The National Health and Nutrition Examination Survey (NHANES) 2007–2016. Missing values for marital status (n = 1,386). Weighted to the U.S. population. Probable depression defined by Patient Health Questionnaire-9 (PHQ-9) cutoff of ≥10.

Fig 3 shows the weighted prevalence of probable depression by race/ethnicity and marital status. Married persons across all race/ethnicity groups had lower probable depression (5.04% for non-Hispanic White, 5.79% for non-Hispanic Black, and 6.67% for Hispanic) than persons who were widowed, divorced or separated (13.08% for non-Hispanic White, 13.13% for non-Hispanic Black, and 15.78% for Hispanic), never married (9.06% for non-Hispanic White, 11.29% for non-Hispanic Black, and 9.73% for Hispanic), or living with partner (9.98% non-Hispanic White, 11.72% non-Hispanic Black, and 10.23% Hispanic).

**Fig 4:**
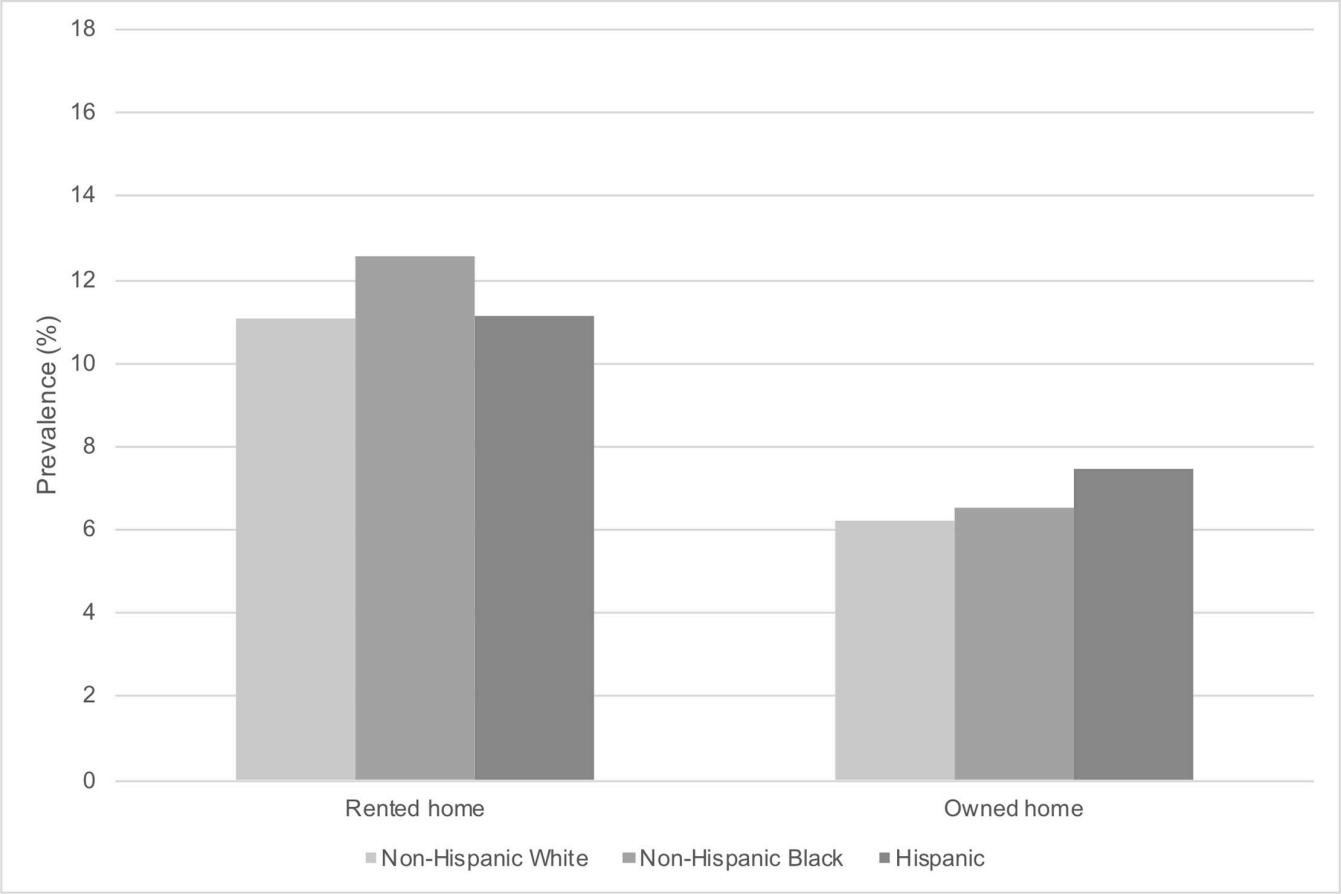
Weighted prevalence of probable depression by race/ethnicity and home ownership in U.S. adults (N = 26,382). The National Health and Nutrition Examination Survey (NHANES) 2007–2016. Missing values for home ownership (n = 295). Weighted to the U.S. population. Probable depression defined by Patient Health Questionnaire-9 (PHQ-9) cutoff of ≥10.

Fig 4 shows the weighted prevalence of probable depression by race/ethnicity and home ownership. Home owners had lower levels of probable depression than home renters across all race/ethnicity groups. Probable depression for home owners ranged from 6.2% (non-Hispanic White) to 6.5% (non-Hispanic Black) to 7.5% (Hispanic). Probable depression for home renters ranged from 11.1% (for both non-Hispanic White and Hispanic) to 12.6% (non-Hispanic Black).

**Fig 5:**
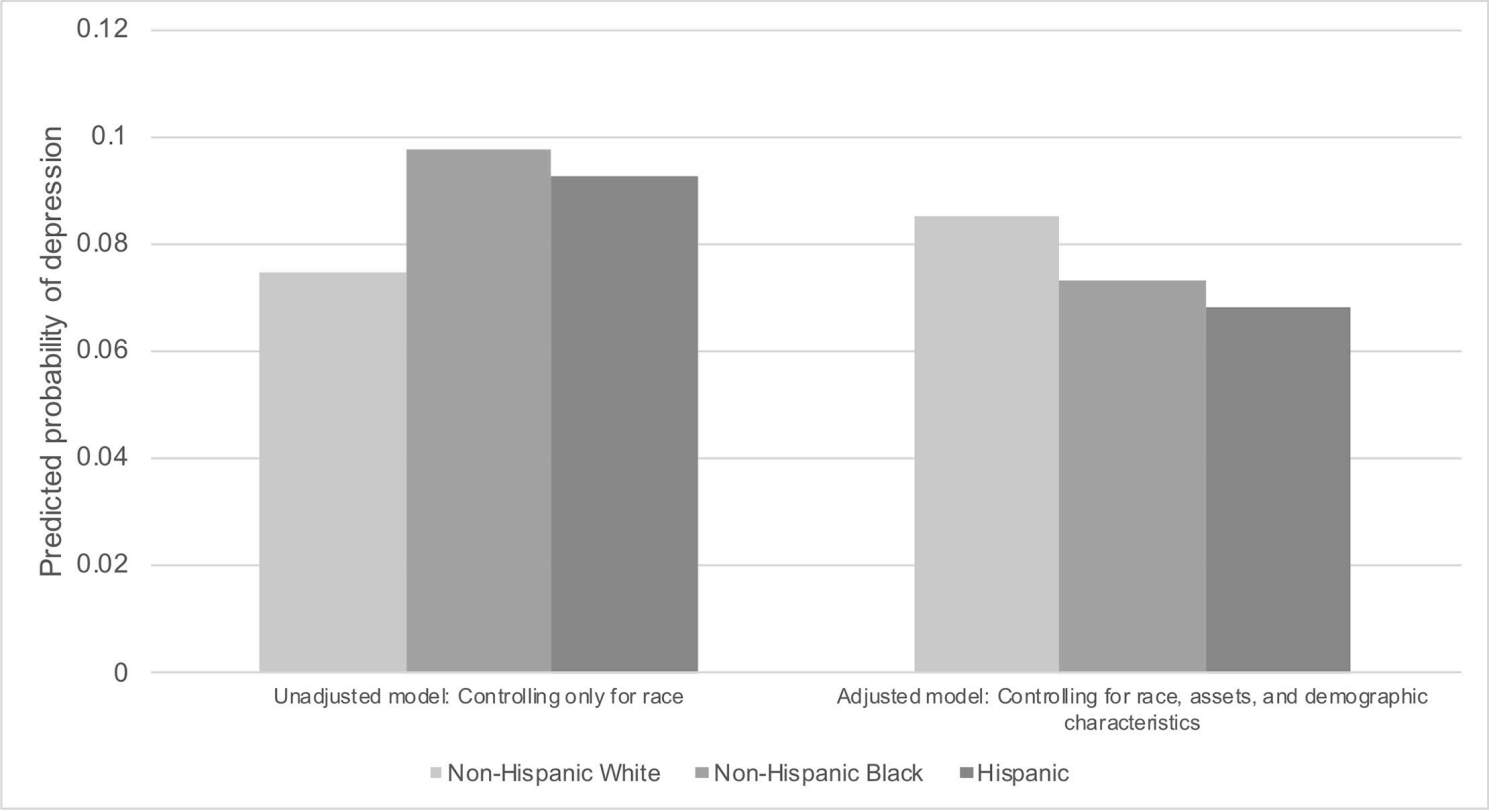
Predicted probability of depression across race/ethnicity using unadjusted and adjusted models in U.S. adults (N = 26,382). The National Health and Nutrition Examination Survey (NHANES) 2007–2016. Weighted to the U.S. population. Unadjusted model controlled only for race/ethnicity and NHANES cohort wave. Adjusted model controlled for race/ethnicity, age, gender, education, marital status, household income, home ownership, household size, and NHANES cohort wave. Probable depression defined by Patient Health Questionnaire-9 (PHQ-9) cutoff of ≥10.

Fig 5 shows the predicted probability of depression across race/ethnicity groups using the unadjusted and adjusted models. The predicted probability of depression was higher for non-Hispanic Black (0.098) and Hispanic (0.092) than or non-Hispanic White (0.074) persons in the unadjusted model and was lower for non-Hispanic Black (0.073) and Hispanic (0.068) than non-Hispanic White (0.085) persons in the fully adjusted model.

## Discussion

In an analysis of a nationally representative sample of U.S. adults, we found that non-Hispanic Black and Hispanic persons had a higher weighted prevalence of probable depression than White persons (9.8% and 9.2% for non-Hispanic Black and Hispanic persons and 7.5% for non-Hispanic White persons). For the three types of assets we studied—financial assets (household income), physical assets (home ownership), and social assets (marital status and education)—we found that the presence of probable depression was lower in higher asset groups across racial and ethnic groups. Thus, having more education, higher income, owning a home, and being married were all associated with lower risk of probable depression regardless of race/ethnicity. Non-Hispanic Black and Hispanic persons had lower levels of assets at every level compared to White persons: they had lower household income and educational attainment and were less likely to be married or own a home. When we looked at risk of probable depression controlling for all assets, we found that non-Hispanic Black and Hispanic persons had a lower risk of probable depression than White persons. Odds ratios and predicted probability of probable depression for non-White persons in adjusted models were lower than for White persons when controlling for assets and demographic characteristics. Thus, lower assets may explain much of the difference in the overall greater prevalence of unadjusted probable depression in non-Hispanic Black and Hispanic persons relative to White persons in the U.S.

One aim of this study was to make sense of the seemingly paradoxical findings in the published literature about race/ethnicity and depression. Our findings are consistent with some studies, and inconsistent with some findings in others, and suggest an explanation for why some studies have documented paradoxical observations for the relation between race/ethnicity and depression.

We found that, overall, non-Hispanic Black and Hispanic persons had a higher weighted prevalence of probable depression than White persons. Prior evidence on this central finding is inconsistent, and this inconsistency was in large part a motivation for our analysis. A recent study of older adults in the U.S. by Peplinski et al. found a higher weighted prevalence of depressive symptoms among Black and Hispanic persons than White persons in unadjusted models [10]. Hudson et al. also reported higher prevalence of depression among Black persons than White persons in current depressive symptoms in a sample of 5,115 Black and White adults ages 18 to 30 years using Coronary Artery Risk Development in Young Adults study (CARDIA) data [9]. Our estimates for unadjusted prevalence of probable depression are consistent with the findings of Peplinski et al. and Hudson et al. who found higher unadjusted prevalence of depression for Black persons relative to White persons. By contrast, our results differ from studies that suggest that Black persons have a lower prevalence of depression [12, 16–18, 34]. Some of these differences may be due to the approach in measuring depression, be it the type of depression measured or the methods used to assess depression. For example, Riolo et al. found that African American persons had a significantly lower lifetime prevalence of MDD than White persons but no significant difference from White persons for lifetime dysthymic disorder, which is a less severe measurement of depression [15]; meanwhile Williams et al. found that lifetime prevalence of MDD was higher among White persons than African American persons, with no difference in 30-day MDD, but with higher chronicity over the lifetime for African American persons relative to White persons [16]. Studies using depressive symptoms were more consistent with our findings [1, 10]; however, Dunlop et al. found similar associations for unadjusted and adjusted prevalence using the more severe Major Depression [11]. It is possible that different patterns emerge when studies use measures of self-report versus clinical diagnoses to measure depression in racial minorities. Our findings do however become consistent with the existing literature once we controlled for assets.

Aiming to more fully explain our findings, we showed that assets were associated with lower depression across all racial and ethnic groups. In particular, income and educational attainment had a dose-response relation with depression. These findings are largely consistent with those of others who have studied race/ethnicity, depression, and income. Peplinski et al. found that higher levels of income were associated with lower odds of depressive symptoms; they found that the association was greatest in White women, Black men, and Hispanic women [10]. Hudson et al. also found a significant relation between income and 12-month depression for African American women [35]. In addition, Hudson et al. found a significant inverse relation between life course SES position and depression for Black and White persons [9]. Further compounding the potential role of assets in explaining racial/ethnic differences in depression, there is a growing literature suggesting that Black persons have lower health gains relative to White persons for higher assets. Assari found that higher income was associated with lower risk of 12-month and 30-day MDD overall but that the reduction in risk, relative to income increase, was smaller for African American than for White persons [36]. While some studies looking at income and race/ethnicity have failed to document the association noted here, there are differences in approaches that explain these inconsistent findings. Assari and Caldwell found that higher income was associated with lower depression for African American girls but higher depression for African American boys. That study however looked only at African American and Caribbean Black adolescents and defined depression as MDD, a more severe diagnosis than our measure [37]. Gavin et al. found that having low income relative to high income was not associated with increased risk of MDD in Black, Latino, or Asian persons; however, that study used Collaborative Psychiatric Epidemiology Studies surveys conducted between 2002 and 2003 and looked at 12-month MDD [12].

We found that greater educational attainment and marital status were also associated with reduced depression across all race and ethnicity groups. The existing literature on the association between education, marital status, and race/ethnicity is divided. Our findings were in line with Lo and Cheng who found improved mental health for higher education, increased income, and marital status [38]. A number of studies have shown, however, that the association between education and marital status on depression may differ by race and gender, which was beyond the scope our analysis. In their CARDIA study, Hudson et al. found that higher household income was associated with greater odds of 12-month Major Depressive Episodes (MDE) among African American men but lower odds of 12-month MDE among African American women. They also found that greater educational attainment was associated with lower odds of 12-month MDE for African American men but had no significant relation on 12-month MDE for African American women. They also found that being married was a significant predictor of reduced depression among African American men but not women [35]. In another study, using data from the National Study of American Life for adults ages 18 and over, Hudson et al. found that neither income nor education were associated with odds of MDD among African American Black, Caribbean Black, or non-Hispanic White persons [10]. Few studies have been done on the relation between home ownership and depression in the U.S. Those studies that have looked to aspects of home ownership and mental health have found that home renters suffer worse mental health than owners and that foreclosure is detrimental to mental health [28, 39].

This study focuses primarily on the relation between probable depression among White and non-Hispanic White persons, in an effort to clarify existing literature and explain the Black-White depression paradox that has been observed. Our finding that Hispanic persons had a higher unadjusted prevalence of probable depression than White persons is consistent with some studies [10, 11] but not others [12]. Previous work has found differences in racial/ethnic groups and depression based on nativity and migration status [40] as well as sub-group membership, which this paper does not cover [41].

After controlling for all three asset categories together, we found that non-Hispanic Black and Hispanic persons had lower risk of probable depression than their White counterparts in the U.S. This is consistent with work that has approached this problem through an assets lens, and supports the Black-White depression paradox. In a study of 7,690 adults aged 54 to 65 in the Health and Retirement Survey, Dunlop et al. found that African Americans had 0.62 (95%CI 0.49–0.79) and Hispanics had 0.92 (95%CI 0.61–1.38) times the odds of developing 12-month major depression relative to whites after controlling for demographic characteristics and economic access, defined as education, income, wealth, health insurance, employment [11]. There are two, rather different, potential explanations for these findings: minorities may be more resilient in the face of mental illness or greater stigma exists in reporting of depressive symptoms. Potential protective factors against mental illness among African Americans include resilience in the face of stressors, strong ethnic identity, or positive attitude [19, 42, 43]. It is also possible that there may be bias in the presentation and diagnosis of depressive symptoms of minority patients, which would suggest under-reporting of depression among those populations, even as studies of the validity of the PHQ-9 suggest it is robust across major racial groups in the U.S. [32, 44].

Taken as a whole, our findings build on a growing literature that looks to foundational causes that may shape racial health inequalities. Bailey et al. offer a conceptual framework for addressing racial health inequalities broadly, which identifies structural racism as a causal factor in contributing to poorer health outcomes for racial minorities through systematically limiting access to housing, education, employment, earnings, benefits, credit, and health care, among other resources [45]. Our paper lends evidence to such frameworks that set out to understand how racial differences in health outcomes came to be. This work also provides data that can inform an intersectional approach to the study of race, mental health, and assets. Patel et al. conducted a systematic review of studies looking at risk and protective factors for depression in racial minority adolescents and suggest that an intersectional lens can help to clarify mechanisms that protect children from depression in different socio-ecological contexts [46]. Jackson et al. propose the concept of a “joint disparity” as a method to assess cumulative disadvantage that racial minorities experience and highlight how outcomes are often patterned across multiple levels of inequality, be they racial or SES groups [47]. Consistent with our study, they suggest that part of racial disparities stem from differential SES (including parental wealth and access to resources from a young age). Nuru-Jeter et al. reviewed the literature looking at race and SES; they note that many studies control for SES and interpret the racial disparities in residual effects remaining as those attributed to race [48]. In our study, the “residual” effect of controlling for SES is actually protective and suggests that racial/ethnic differences in depression outcomes may be largely attributed to access to assets. In this way, this body of work suggests that there is a cumulative association between race/ethnicity and mental health, and that financial disparities may play a large role in driving disparate health outcomes between racial/ethnic groups.

There is a growing literature on diminishing returns of resources for Black relative to White persons, generally called the *minorities’ diminished returns* theory [49]. In our study, within all but two asset groups (household income of $0-$19,000 and $45,000-$74,999), Black persons did appear to have a higher prevalence of depression than White persons. Other authors have written on the subject, suggesting that racial discrimination and other factors contribute to reduced health gains for Black persons relative to White persons [12]. A review of reviews on health outcomes for Black persons relative to White persons suggests that assets other than income appear to produce a smaller yield in health gains for Black persons than White persons; however, they conclude that income does appear to improve outcomes similarly, suggesting the importance of focusing on all assets and income in particular to improve minority health outcomes [36, 50]. A fuller analytic exploration of this phenomenon was beyond the scope of our analysis, but suggests potential fruitful directions for future work.

There are three principal limitations to this study. First, this analysis used cross-sectional data, which measured probable depression at one point in time. It should be noted that there may be a bidirectional relation of assets and depression; persons with depression may have difficulties acquiring or keeping assets [2]. Second, the instrument for capturing depression in NHANES, the PHQ-9, was based on self-report. A fuller assessment of clinical depression would require physician diagnosis. Third, due to the definition of race/ethnicity in NHANES questionnaires from 2007–2010, we cannot disaggregate non-Hispanic Asian from Other race or multiracial persons. Due to differential health outcomes and exposures to various assets across these groups, the findings for these categories may be less meaningful to interpret.

## Conclusions

Notwithstanding these limitations, this study adds three findings to the literature. First, these findings show that non-Hispanic Black and Hispanic adults have a higher prevalence of probable depression in the U.S. than non-Hispanic White persons. Second, we found that assets are associated with lower prevalence of probable depression in all categories across all race/ethnic groups. We also found that Black and Hispanic persons had lower assets than White persons in all categories studied: financial assets (household income), physical assets (home ownership), and social assets (marital status and education). Third, we found that once we accounted for differences in assets, non-Hispanic Black and Hispanic persons had a lower risk of probable depression than their White counterparts. These findings suggest that minorities may be more resilient in the face of mental illness when assets are taken into account or that minorities face greater stigma in reporting symptoms of depression than White persons. Future research may wish to further explore how access to additional assets such as family wealth or savings may protect against mental illness across racial/ethnic groups.
